# LCN2‐ACOD1 Signalling Affects the Post‐Injury Regeneration of Skeletal Muscle Through Mediating Ferroptosis

**DOI:** 10.1111/cpr.70130

**Published:** 2025-09-22

**Authors:** Xiaojing Hao, Hongwei Shi, Di Wu, Rui Liang, Tong Zhao, Wen Sun, Yue Wang, Xiuju Yu, Xiaomao Luo, Yi Yan, Jiayin Lu, Haidong Wang, Juan Wang

**Affiliations:** ^1^ College of Veterinary Medicine Shanxi Agricultural University Taigu Shanxi People's Republic of China; ^2^ Department of Nephrology, Shanghai General Hospital Shanghai Jiao Tong University School of Medicine Shanghai People's Republic of China

**Keywords:** ACOD1, ferroptosis, LCN2, mitochondria, skeletal muscle regeneration

## Abstract

The normal growth and development of skeletal muscle are crucial for the proper function of organisms. During myoblast development, cell death is a fundamental physiological process, and skeletal muscle damage involves various types of cell death, including ferroptosis. However, ferroptosis‐related biomarkers in skeletal muscle damage remain unclear. This study aimed to investigate the mechanisms by which lipocalin‐2 (LCN2), a key protein of iron metabolism, regulates skeletal muscle regeneration post damage by mediating ferroptosis. When the gastrocnemius muscle (GAS) of mice is acutely injured, LCN2 is significantly upregulated early in the injury. In vitro, LCN2 participates in the inhibition of proliferation and differentiation of C2C12 cells via erastin‐induced ferroptosis. Transcriptomic analysis after the overexpression of LCN2 revealed that the one with the most significant difference among all of the differentially expressed genes (DEGs) was aconitate decarboxylase 1 (Acod1). The inhibition of myogenic factors' expression by LCN2 was associated with the activation of the ferroptosis signalling pathway, partly attributed to the mitochondrial dysfunction. The ACOD1 inhibitor attenuated mitochondria‐associated ferroptosis induced by LCN2 and alleviated the inhibitory effect of LCN2 on cell viability. These findings highlight the therapeutic potential of targeting the LCN2‐ACOD1 signalling to promote myogenesis, providing promising strategies for facilitating the regeneration of skeletal muscle after injury and the treatment of muscle‐related diseases.

## Graphical abstract

LCN2 mediates the suppression of C2C12 myoblast proliferation and myotube formation via erastin‐induced ferroptosis. Mechanistically, the LCN2‐ACOD1 axis regulates skeletal muscle growth via mitochondria‐associated ferroptosis, providing a theoretical basis for the prevention and therapy of muscle‐related diseases.

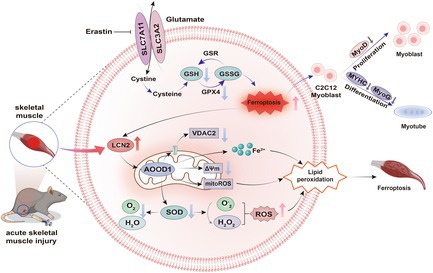

**Abbreviations**
ACOD1: aconitate decarboxylase 1
ACSL4: long‐chain fatty acyl‐CoA synthetase 4
BCA: bicinchoninic acid
CMAS: N‐acylneuraminate cytidylyltransferase
CSA: cross‐sectional area
DEGs: differentially expressed genes
Era: erastin
FBS: foetal bovine serum
Fer‐1: ferrostatin‐1
Fth1: ferritin heavy chain
FTL: ferritin light chain
GAS: gastrocnemius
GO: gene ontology
GPX4: glutathione peroxidase 4
GSEA: gene set enrichment analysis
H&E staining: haematoxylin and eosin staining
Hmox1: haeme oxygenase 1
IF: immunofluorescence
KEGG: Kyoto Encyclopedia of Genes and Genomes
LCN2: lipocalin‐2
MDA: malondialdehyde
MMP: mitochondrial membrane potential
Myh3: myosin heavy chain 3
MyoD: myoblast determination
MyoG: myogenin
NCOA4: nuclear receptor coactivator 4
Pax7: paired box 7
RIPA: radio immunoprecipitation assay
ROS: reactive oxygen species
SLC7A11: solute carrier family 7 member 11
SOD: superoxide dismutase
TEM: transmission electron microscopy
VDAC2: voltage‐dependent anion channel 2

## 1 Introduction

Skeletal muscle is an important organ for supporting body structure, controlling movement and storing energy [1]. As part of the cellular life cycle, cell death is a fundamental physiological process in myoblasts' development that extends from embryonic development, organ maintenance, ageing to the coordination of immune response and autoimmunity [2]. Typically, a variety of physiological processes in skeletal muscle are in a homeostatic state, partly owing to the precise regulation of cell death. However, when skeletal muscle is damaged, apoptosis, necrosis and autophagy coexist to affect the regeneration of skeletal muscle homeostasis and integrity [3], in addition to the recently discovered ferroptosis. Ferroptosis has emerged as a hotspot due to its involvement in the development of a plethora of diseases, such as cancers [4], cardiovascular diseases [5], liver‐related diseases [6] and degenerative diseases [7]. In addition, increasing reports suggest a strong link between ferroptosis and skeletal muscle damage and myopathy [8, 9], which indicates that targeting ferroptosis to regulate skeletal muscle regeneration has broad implications.

Ferroptosis is characterised by severe lipid peroxidation initiated by iron overload and the generation of reactive oxygen species (ROS) [10], in which intracellular iron levels are determined by iron uptake, storage, release and metabolism [11]. The key protein of iron metabolism, lipocalin‐2 (LCN2, also known as siderocalin, 24p3), binds Fe^2+^ (holo‐24p3) and transports it intracellularly via endocytosis, as well as entering the cytosol to bind Fe^2+^ and transport it extracellularly through an independent form (apo‐24p3) [12]. As a regulator of ferroptosis, LCN2 plays an important role in a variety of diseases, such as lung cancer [13], colorectal cancer [14] and central nervous system diseases [15]. In recent years, the role of LCN2 in muscle metabolism has been revealed. Related studies have shown that LCN2 is a regulator of regeneration after skeletal muscle injury [16]. However, few studies have elucidated the specific mechanisms by which LCN2 regulates the post‐injury regeneration of skeletal muscle by mediating ferroptosis. In this study, RNA sequencing was performed after LCN2 was overexpressed in C2C12 myoblasts, which were subsequently screened to obtain the one with the most significant difference among all of the differentially expressed genes (DEGs), namely, aconitate decarboxylase 1 (ACOD1).

ACOD1 mediates itaconate production, oxidative stress, antigen processing and plays dual roles in immunity and diseases [17]. ACOD1 is found in mitochondria [18, 19] and has been shown to regulate ROS production derived from mitochondria as well as intracellularly [18]. These findings indicate dual roles of ACOD1‐mediated oxidative stress via the regulation of ROS production in shaping the immune response [19, 20, 21]. Furthermore, since ROS‐mediated lipid peroxidation is a key factor in the process of ferroptosis [22], it is hypothesised that ACOD1, a downstream signalling molecule of LCN2, could regulate skeletal muscle regeneration post‐injury by mediating mitochondria‐associated ferroptosis.

In our study, BaCl_2_ was injected into the gastrocnemius (GAS) muscle to establish an acute skeletal muscle injury model. Our findings identified a previously unrecognised function of LCN2‐ACOD1 signalling in the process of ferroptosis in skeletal muscle, which is important for understanding the regulation of the regeneration process of damaged skeletal muscles.

## 2 Materials and Methods

### 2.1 Animals

The study was approved by the Institutional Animal Care and Use Committee of Shanxi Agricultural University [SXAU‐EAW‐2023 M.ZZ.004009196]. 8‐week‐old male C57BL/6J mice were purchased from SPF (Beijing) Biotechnology Co. Ltd. The animals were housed at 22°C ± 3°C in a specific pathogen‐free facility (12 h/12 h light/dark cycle, 40%–50% humidity) and ventilated under specific pathogen‐free conditions with ad libitum access to food and water. The mice were acclimatised for 1 week. To construct a model of acute skeletal muscle injury in mice, they were anaesthetised with ether before the GAS muscle was locally sterilised and injected with a 1.2% BaCl_2_ solution. After 1 day (1 d), 3 days (3 d), 5 days (5 d), 7 days (7 d) and 9 days (9 d) after injury, healthy and injured mice were euthanised to collect blood and GAS. A portion of GAS was fixed, and the remaining was stored in liquid nitrogen for subsequent molecular tests.

### 2.2 Cell Culture and Treatment

C2C12 cells were cultured in growth medium, which consisted of Dulbecco's modified Eagle's medium (DMEM, 25 mM glucose, Cytiva, Uppsala, Sweden), 10% foetal bovine serum (FBS) (YoBiBi, Shanghai, China) and 1% penicillin–streptomycin (Solarbio, Beijing, China). When the cells reached 80% confluence, the growth medium was changed to differentiation medium, which consisted of DMEM supplemented with 2% horse serum (Solarbio, Beijing, China) and 1% penicillin–streptomycin. The cells were used for experiments until the myotubes were essentially fully fused. All cells were maintained in a humidified atmosphere of 95% air and 5% CO2 at 37°C and the DM was changed every day.

Erastin (era), ferrostatin‐1 (Fer‐1) (Sigma‐Aldrich, E7781 and SML0583, Missouri, USA) and ACOD1 inhibitor (ACOD1‐IN) (MCE, HY‐148335, New Jersey, USA) were dissolved in dimethylsulphoxide (DMSO) according to the specification. After concentration screening, the working concentrations of era, Fer‐1 and ACOD1‐IN were 5 μM, 10 μM and 20 μM respectively. In the subsequent experiments, the C2C12 myoblasts and myotubes were treated for 48 h, and the differentiating C2C12 cells were treated for 5 days.

### 2.3 Histological Analysis

After the euthanasia of healthy and injured mice, a portion of GAS tissue was fixed in 4% paraformaldehyde for 30 h to prepare 6 μm paraffin sections of GAS cross sections, followed by staining with haematoxylin and eosin (H&E) staining and Masson staining according to the manufacturer's instructions. Whether the acute skeletal muscle injury model was successfully constructed can be initially identified through histological observations and statistical analyses of images by a microscope (Nikon Eclipse Ts2R, Japan) equipped with NIS‐Elements imaging software.

### 2.4 Western Blot Analysis

Skeletal muscle tissues and cells were lysed with radio immunoprecipitation assay (RIPA) lysis buffer (Beyotime, P0013B, Shanghai, China) containing PMSF protease inhibitor (Servicebio, G2008, Wuhan) for 30 min and then centrifuged at 14,000 × g for 5 min to obtain total protein. The total protein concentration was determined with a bicinchoninic acid (BCA) protein assay kit (Beyotime, P0011, Shanghai, China). Aliquots of proteins (loading quantity of protein sample: 40 μg) were separated by SDS‐PAGE, transferred to a PVDF membrane (Millipore, ISEQ00010, MA, USA) and blocked with 5% skim milk powder (Absin, abs9175, Shanghai, China) for 2 h. Membranes were incubated with specific primary antibodies (Table S1) at 4°C overnight. After being washed in TBST, the PVDF membranes were incubated with the secondary antibody in a shaker at 37°C for 1 h. The target protein bands were obtained by using a high‐sensitivity chemical imager. Finally, protein bands were detected using ChemiDOC XRS+ Imaging System (Bio‐Rad, Universal Hood II, CA, USA) and visualised via the Image Lab 4.0.1 system.

### 2.5 Quantitative Reverse Transcription‐Polymerase Chain Reaction (qRT‐PCR)

Total RNA was extracted with RNAiso Plus (Takara, 9109, Kyoto, Japan) and reverse‐transcribed to cDNA via a reverse transcription kit (Vazyme, R223‐01, Nanjing, China). The primer sequences were designed using PrimerBank, and primer synthesis was performed by Shanghai General Biological Company (Shanghai, China). QRT‐PCR amplification was performed using the MonAmp SYBR Green qPCR mix (Monad, MQ10201, Jiangsu, China). The results were calculated from the ^ΔΔ^CT values. The primers used are shown in Table S2.

### 2.6 Transmission Electron Microscopy (TEM)

After the BaCl_2_ solution was injected into the GAS, the GAS of healthy and injured mice for 3 d were immersed in 2.5% glutaraldehyde fixative (Solarbio, G1102, Beijing, China) pre‐cooled at 4°C in a size of 1 mm × 1 mm × 2 mm. After osmium fixation, dehydration, permeabilisation and embedding, sections were obtained at 70–90 nm, which were stained with peroxide acetate (15 min) and lead citrate (5 min), dried and then examined for mitochondrial morphology by TEM (JEOL, JEM1200EX, Tokyo, Japan).

### 2.7 Serum Iron Assay

Blood was collected, placed at room temperature for 1 h to ensure complete coagulation, and then centrifuged at 3000 × g (4°C) for 15 min to collect the supernatant for serum iron content measurement. According to the manufacturer's instructions (Jiancheng Bio, A039‐1‐1, Nanjing, China), double‐distilled water and iron standard application solutions were used as a negative control and a positive control, respectively. Iron colour developer was added to the control and serum groups. The above mixtures were mixed, respectively, boiled (5 min), cooled and centrifuged at 3500 r/min for 10 min, and the supernatant was collected and read at 520 nm with an enzyme meter.

### 2.8 Fe^2+^ Content

To detect the ferroptosis effect in C2C12 myoblasts and myotubes, the cells were treated with specific drugs for 48 h and then the FerroOrange probe (DOJINDO, F374, Kumamoto, Japan) was used for the detection of Fe^2+^. The concentration of FerroOrange working solution was diluted to 1 mmol/L with serum‐free medium. The cells were washed with DMEM and incubated in the cell culture incubator for 30 min. The cells were observed under a fluorescence microscope and photographed.

### 2.9 Assessment of Antioxidant Status and Lipid Peroxidation

GAS tissues or C2C12 cells were lysed in RIPA, total protein was obtained by centrifugation of the supernatant, and the protein concentration was measured with a BCA kit. The malondialdehyde (MDA) content and superoxide dismutase (SOD) activity were measured respectively and normalised to protein concentration (μmol/mg protein for MDA; U/mg protein for SOD) according to the kit manufacturer's instructions (Beyotime, S0131S and S0101S, Shanghai, China). Afterwards, values were calculated via the optical density of MDA at 532 nm and the optical density of SOD at 450 nm.

The intracellular levels of ROS in C2C12 cells cultured in 48‐well plates were assayed using a commercial assay kit (Beyotime, S0033S, Shanghai, China). Rosup solution (50 mg/L) was used as a positive control to stimulate the cells for 30 min, after which the cells were loaded with a DCFH‐DA probe (10 μmol/L) in situ and incubated for 30 min in the cell culture incubator and then photographed under a fluorescence microscope (excitation wavelength: 488 nm, emission wavelength: 525 nm).

After C2C12 cells were treated with or without ACOD1‐IN after overexpression or non‐overexpression of LCN2 for 48 h, the superoxide content in the mitochondria was detected by MitoTracker Green (Beyotime, C1048, Shanghai, China) and MitoSOX (DOJINDO, MT14, Kumamoto, Japan). MitoTracker Green and MitoSOX were dissolved in DMSO solution. To localise the mitochondria, the cells were incubated at 37°C with prewarmed MitoTracker Green at a final concentration of 150 nM for 1 h in a cell culture incubator and then treated with 10 μmol/L MitoSOX for 30 h. After the working solution was discarded, images were taken under a confocal microscope (Olympus, FV1000, Tokyo, Japan) (excitation wavelength of 490 nm and emission wavelength of 516 nm for MitoTracker, excitation wavelength of 633 nm and emission wavelength of 700 nm for MitoSOX).

### 2.10 Mitochondrial Membrane Potential (MMP, Δψm) Detection

The MMPs of myoblasts cultured in 96‐well plates were analysed using the JC‐1 probe (Beyotime, C2003S, Shanghai, China). In normal cell mitochondria, JC‐1 aggregates and shows bright red fluorescence (excitation wavelength of 525 nm and emission wavelength of 590 nm). However, when the MMP is decreased, JC‐1 exists in the form of monomers, and the green fluorescence is significantly enhanced (excitation wavelength of 490 nm and emission wavelength of 530 nm). The JC‐1 working solution was mixed thoroughly, and the cells were treated for 30 min in a cell culture incubator at 37°C. The cells were terminated by loading the probes, washed and photographed using the fluorescence microscope and analysed using Image J.

### 2.11 Cellular Immunofluorescence (IF) Staining

Cellular IF staining was performed by incubating antibodies following fixation, permeabilisation and blocking. IF staining of myotubes antibodies included MYHC (Proteintech, 22,287–1‐AP, Wuhan, China), and secondary antibodies Goat Anti‐Rabbit IgG H&L (abcam, ab150077, Cambridge, England). The fusion index was calculated by dividing the number of nuclei contained within multinucleated cells by the number of total nuclei in a field. Photographed using a fluorescence microscope, the fusion index and the diameter of myotubes were analysed using Image J software.

The co‐localisation of ACOD1 and mitochondria after the C2C12 cells were fixed with 4% paraformaldehyde for 15 min. After a 1‐h pre‐incubation with the Mitotracker Green dye as described in 2.9, the above cellular IF staining operation was carried out, and images were taken using a confocal microscope.

### 2.12 Plasmid Extraction and Transfection

Mouse LCN2 was cloned and inserted into the pcDNA3.1+ vector, and the plasmids of pcDNA3.1+ (NC) and pcDNA3.1 + ‐LCN2 (LCN2) plasmids were extracted by the EndoFree Mini Plasmid Kit (TIANGEN, DP118‐02, Beijing China), and then transfected into cells using liposome‐mediated transfection. C2C12 myoblasts were cultured in 6‐well and 96‐well plates with 3 μg and 100 ng of plasmids per well, respectively, and 5 μL and 0.2 μL of Lipofectamine 2000 reagent (Invitrogen, 15,338,100, Thermo Fisher Scientific, MA, USA), respectively, and transfected with DMEM for 6 h. Then, the medium was switched to medium containing 10% FBS for 48 h.

### 2.13 Gene Knockdown by siRNA

C2C12 cells were seeded in 6‐ or 96‐well plates. When the density reached 50%, the complex diluted in serum‐free DMEM was transfected into C2C12 cells, including Lipofectamine 2000 reagent and the si‐RNA oligonucleotides targeting LCN2. After 6 h, the medium was changed to growth medium and the cells were cultured for 48 h before subsequent experiments were conducted. Si‐NC was used as the negative control. The synthesis of si‐RNAs was completed by Shanghai General Biological Company (Shanghai, China). The nucleotide sequences of si‐RNAs are as follows: si‐NC, sense (5′‐3′): UUC UCC GAA CGU GUC ACG UTT, antisense (5′‐3′): ACG UGA CAC GUU CGG AGA ATT; si‐LCN2, sense (5′‐3′): GCA CAG GUA UCC UCA GGU ATT, antisense (5′‐3′): UAC CUG AGG AUA CCU GUG CTT.

### 2.14 RNA Sequencing (RNA‐Seq) and Bioinformatics Analysis

At the end of 48 h of plasmid transfection, total RNA from NC and LCN2 groups was isolated by Trizol reagent. PCR library amplification was performed using Hieff NGSUltima Dual‐mode mRNA Library Prep Kit (Yeasen, 12309ES, Shanghai, China). The libraries were then sequenced on the Illumina NovaSeq X Plus platform. The expression abundance and variations for each of the genes were normalised to fragments per kilobase of transcript per million mapped reads (FPKM) using RNA‐seq by expectation maximisation (RSEM). Our criteria for screening significant DEGs were log_2_^FC^>log_2_^1.5^ and P‐value < 0.05. In addition, we performed cluster analysis and gene set enrichment analysis (GSEA) for DEGs. GSEA focused on gene ontology (GO) and Kyoto Encyclopedia of Genes and Genomes (KEGG). Extended protocol details and procedures for data analysis were provided by GENE DENOVO (Guangzhou, China).

### 2.15 Statistical Analysis

Experiment results are presented as means ± standard errors of the means (SEMs) of at least three replicates and were analysed with GraphPad Prism 9.4.1 software (GraphPad Software, San Diego, USA). Student's two‐tailed t‐test (unpaired) was performed to determine significant differences. Values were considered statistically different at *p* < 0.05. Differences with *p* < 0.05 were considered significantly different. *p*‐values are presented as follows: *p < 0.05, **p < 0.01, ***p ≤ 0.001, ^#^ *p* < 0.05, ^##^ *p* < 0.01, ^###^ *p* ≤ 0.001 and ns: not significant (*p* ≥ 0.05).

## 3 Results

### 3.1 BaCl_2_ Activates Acute Injury and Regeneration of Skeletal Muscle

The results suggest that the injection of BaCl_2_ for 1 d was accompanied by the beginning of the lysis of the injured myocytes and the appearance of inflammation (Figure S1A). The muscle fibres suffered the most damage on 3 d, as the largest number of smaller‐sized muscle fibres and the smallest cross‐sectional area (CSA) were observed (Figure S1B,C). Neutrophils rapidly invade the damaged areas, followed by speedy differentiation of monocytes into macrophages. Infiltration peaked on 3 d after and progressively decreased. Macrophages exerted dominant functions among inflammatory cells to promote injury repair, including phagocytosing damaged muscle tissue and cell debris, producing pro‐inflammatory and anti‐inflammatory cytokines to regulate inflammation [23]. Myofibers containing two or more centrally located nuclei involved in the regeneration were prominently detectable on 5 d, and the tissue basically healed on 9 d after injury (Figure S1D). Fibroblasts in the connective tissue began to synthesise and secrete collagen fibres from 1 d to 3 d of injury. By 5 d, collagen fibre synthesis reached its peak, followed by gradual recovery to rebuild the damaged muscle tissue (Figure S1E,F). The expression levels of muscle development markers, namely, paired box 7 (Pax7), myoblast determination (MyoD) and myogenin (MyoG) were gradually increased with the injury of GAS and then decreased to normal levels with regeneration (Figure S1G–I). These data indicate that BaCl_2_ activates a coordinated process of tissue degradation and regeneration, which is consistent with previous findings [24, 25, 26].

### 3.2 Involvement of Ferroptosis in the Skeletal Muscle Injury Model and the Expression Pattern of LCN2

**FIGURE 1:**
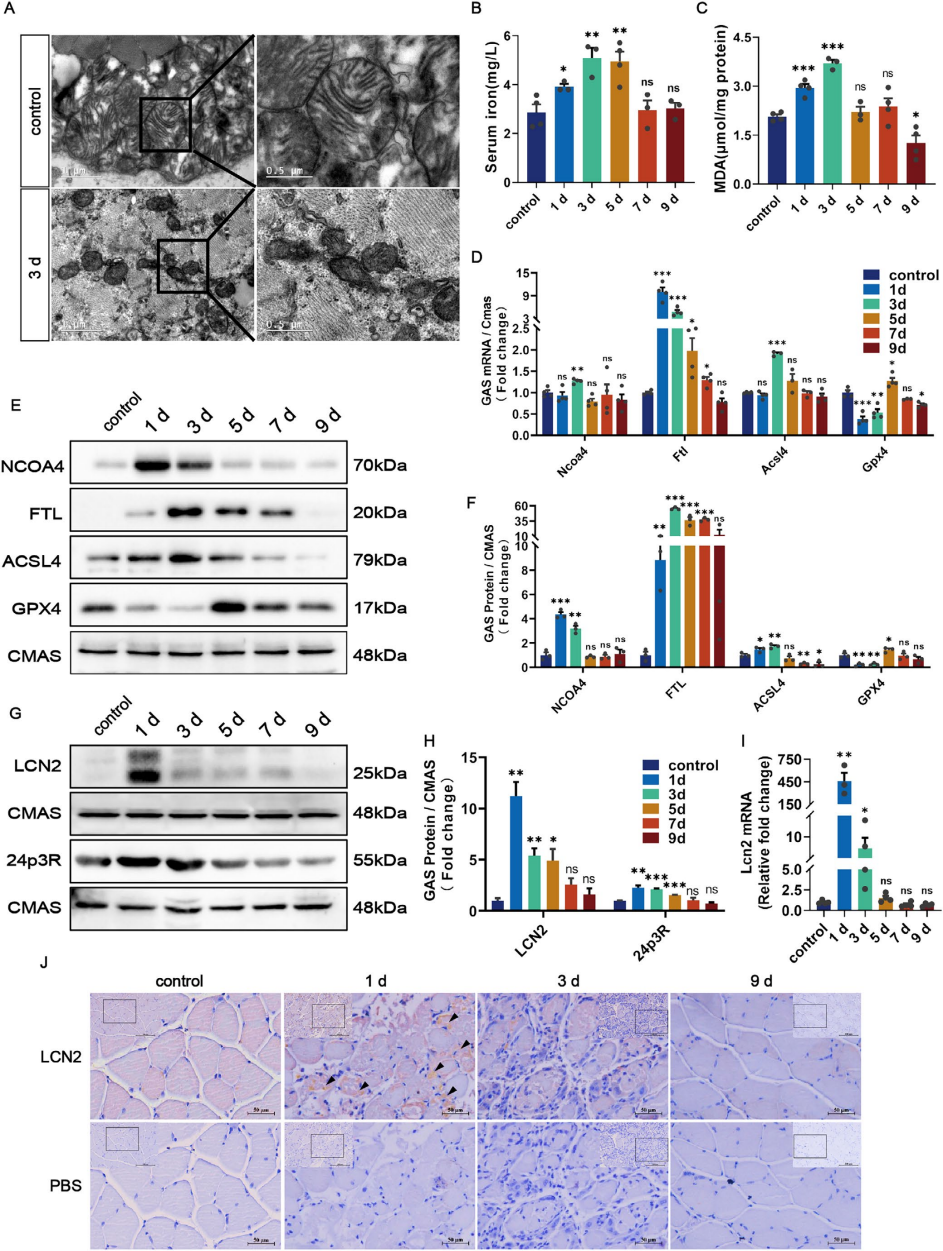
Ferroptosis is involved in skeletal muscle injury and the expression pattern of LCN2. (A) Transmission electron microscopy images for mitochondria of GAS sections from mice before and after BaCl_2_‐induced injury for 3 days. Scale bars = 0.5 μm. (B, C) Determination of serum iron and MDA contents of GAS at different time points before and after injury. (D–F) QRT‐PCR analysis of *Ncoa4, Ftl, Acsl4* and *Gpx4* mRNA and Western blots were used to detect the same protein expression of GAS from mice before and after BaCl_2_‐induced injury. (G–I) QRT‐PCR analysis of *Lcn2* mRNA and levels of LCN2 and 24p3R proteins were assessed by Western blots. (J) Representative immunohistochemistry images of LCN2 of GAS from mice before or after BaCl_2_‐induced injury on 1, 3 and 9 days (black arrows mark positive expression sites of LCN2) Scale bars = 200 μm and 50 μm. Cmas was used for qRT‐PCR normalisation, and CMAS was used as a loading control for the Western blots. ns, not significant, (*p < 0.05, **p < 0.01 and ***p < 0.001) relative to the control, two‐sided Student's t‐test was used. Data represent the means ± SEMs (*n* = 3–4 per group).

Morphologically, compared with normal mitochondria, mitochondria from skeletal muscle subjected to the most severe damage on 3 d presented typical ferroptosis features [27], such as greater membrane density, a smaller volume, and a reduction in or even the disappearance of cristae (Figure 1A). Mechanistically, the serum Fe^2+^ content and the expression levels of iron metabolism‐related indices nuclear receptor coactivator 4 (NCOA4) and ferritin light chain (FTL) were significantly upregulated on 3 d postinjury, meaning that injury increased free iron in the iron pool by promoting ferritin degradation (Figure 1B,D–F). The content of lipid peroxides MDA and the expression of lipid metabolism enzyme long‐chain fatty acyl‐CoA synthetase 4 (ACSL4), which accelerates lipid peroxide generation [28], were significantly increased on 3 d postinjury (Figure 1C–F). Glutathione peroxidase 4 (GPX4), an antioxidant indicator, was significantly downregulated on 1 d and 3 d after injury and significantly upregulated by 5 d to participate in repair (Figure 1D–F). Upon the completion of repair, the above indices gradually returned to normal levels.

In addition, the expression level of LCN2, a key factor associated with ferroptosis, was rapidly increased on 1 d after injury and preceded changes in ferroptosis‐related indices, followed by a gradual abatement. The receptor 24p3R of LCN2 is consistent with the changes in LCN2 (Figure 1G–I). Furthermore, we observed that LCN2 was mainly localised in damaged myofibers (Figure 1J). Thus, ferroptosis is involved in skeletal muscle injury in mice and the involvement is highest during the most severe period of injury, while the changes in LCN2 are upstream of the onset of ferroptosis.

### 3.3 LCN2 Participates in the Inhibition of C2C12 Myoblasts Proliferation by Ferroptosis

**FIGURE 2:**
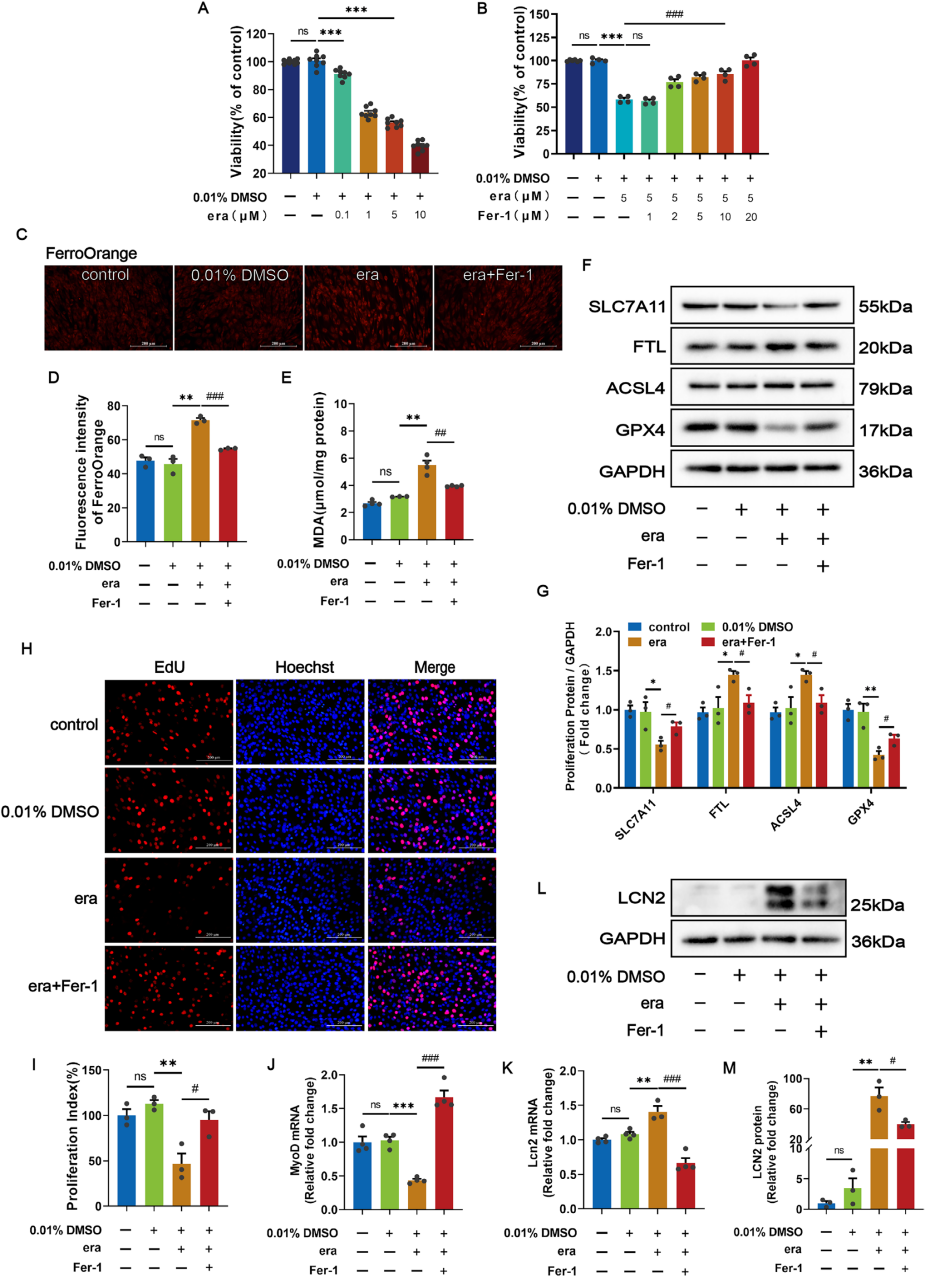
LCN2 Participates in the inhibition of C2C12 myoblast proliferation by ferroptosis. (A) Cell viability of C2C12 myoblasts treated with different concentrations of erastin for 48 h. (B) Cell viability with 5 μM erastin either alone or in the presence of different concentrations of Fer‐1 up to 48 h. (C, D) Representative images showing FerroOrange fluorescent probe labelling of Fe^2+^ content in the indicated cells and their quantitative analysis. Scale bars = 200 μm. (E) Detection of lipid peroxide (MDA) content. (F, G) Western blots of SLC7A11, FTL, ACSL4 and GPX4 expression and quantification in proliferating C2C12 myoblasts following incubation with 5 μM erastin either alone or in the presence of 10 μM Fer‐1 up to 48 h. (H, I) EdU staining and analysis of cell proliferation index verified that ferroptosis represses the proliferation of C2C12 myoblasts. Scale bars = 200 μm. (J) QRT‐PCR analysis of the inhibition of MyoD mRNA expression following the occurrence of ferroptosis. (K) QRT‐PCR analysis of LCN2 mRNA expression in C2C12 myoblasts following ferroptosis. (L, M) Representative images and analysis of LCN2 protein in C2C12 myoblasts following the occurrence of ferroptosis. β‐Actin was used for qRT‐PCR normalisation, and GAPDH was used as a loading control for the Western blots. ns, not significant, (*p < 0.05, **p < 0.01 and ***p < 0.001) relative to the 0.01% DMSO group, (^#^ *p* < 0.05, ^##^ *p* < 0.01 and ^###^ *p* < 0.001) relative to the era group, as determined by two‐sided Student's t‐test. The data represent the means ± SEMs (*n* = 3 per group).

To clarify the effects of ferroptosis on the proliferation of myoblasts, the ferroptosis activator erastin was used to induce ferroptosis of C2C12 myoblasts. Compared with the 0.01% DMSO group, CCK‐8 results showed that the viability of C2C12 myoblasts was negatively correlated with the concentration of erastin, and 5 μM erastin was chosen for further cell experiments (Figure 2A). C2C12 myoblasts were subsequently treated with 5 μM erastin either alone or in the presence of ferrostatin‐1, a ferroptosis inhibitor at concentrations ranging from 1 to 20 μM (Figure 2B). Fer‐1 dose‐dependently reversed the inhibitory effect of erastin on cell viability. Based on our existing research experience, we selected 10 μM Fer‐1 for the subsequent experiments. After erastin stimulation, the Fe^2+^ content and FTL expression level in C2C12 myoblasts were significantly increased, suggesting free iron accumulation (Figure 2C,D,F,G). FerroOrange is a fluorescent probe that specifically detects unstable iron (II) ions (Fe^2+^) [29]. The downregulation of solute carrier family 7 member 11 (SLC7A11) and GPX4 expression suggests the disruption of anti‐lipid peroxidation to drive ferroptosis (Figure 2F,G). The MDA content and ACSL4 expression were increased, indicating disturbances in redox homeostasis and the overload of lipid peroxidation (Figure 2E–G). However, these changes were reversed by Fer‐1 supplementation (Figure 2C–G). These data indicate that erastin induces ferroptosis in C2C12 myoblasts.

EdU staining was used to track proliferating cells, showing that erastin‐induced ferroptosis decreased the proportion of EdU‐positive cells (Figure 2H,I) and MyoD mRNA levels (Figure 2J). Interestingly, LCN2 expression levels were increased in erastin‐treated proliferating C2C12 myoblasts (Figure 2K–M). Taken together, the proliferation of C2C12 myoblasts is inhibited when erastin‐induced ferroptosis occurs, and LCN2 is involved in the process mentioned above.

### 3.4 LCN2 Is a Crucial Factor in Attenuating Myogenic Differentiation of C2C12 Myoblasts Through Ferroptosis

When the cell density reached 80%, the growth medium (GM) was replaced with a differentiation medium (DM) containing 5 μM erastin with or without inhibitors Fer‐1. The medium containing the chemicals was updated once a day. Five days after cell differentiation, Fe^2+^, ROS and lipid peroxidation product MDA in the cells were significantly upregulated when ferroptosis was activated (Figure S2A–E). In addition, a significant decrease in SLC7A11 in response to erastin resulted in the suppression of GPX4 expression. Upregulation of ACSL4 ultimately triggered ferroptosis by promoting lipid peroxidation to disrupt redox homeostasis (Figure S2F,G). These can be rescued by Fer‐1.

Cellular immunofluorescence (IF) staining of MYHC performed on cells differentiated for 1, 3 and 5 days revealed that significantly fewer cells would be differentiating upon activation of ferroptosis, implying that fewer cells were available for myotube fusion (Figure S2H,I). The fusion index as well as the diameter of myotubes was also significantly reduced in myoblasts that had been differentiated for 5 days (Figure S2J,K). All of these effects could be rescued by Fer‐1. Similar to proliferating myoblasts, LCN2 is significantly upregulated in differentiating myoblasts when ferroptosis is activated (Figure S2L,M). It can be concluded that LCN2 correlates with impaired differentiation myogenic differentiation of C2C12 myoblasts through ferroptosis.

### 3.5 LCN2 Is Involved in the Inhibition of C2C12 Myotube Formation by Ferroptosis

**FIGURE 3:**
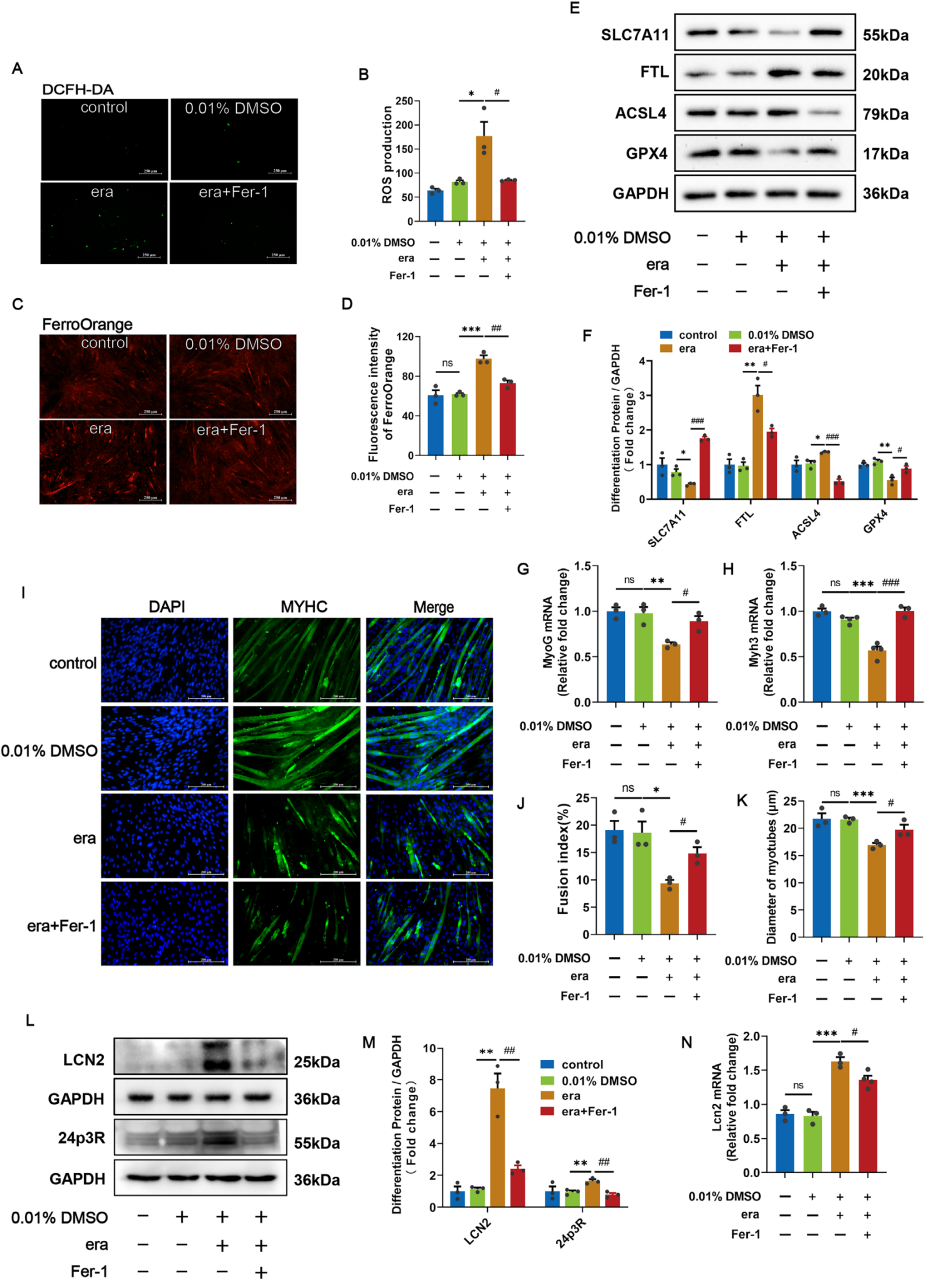
LCN2 Is involved in the inhibition of C2C12 myotube formation by ferroptosis. After 5 days of differentiation, C2C12 myotubes were treated with 5 μM erastin either alone or in the presence of 10 μM Fer‐1. (A, B) Representative images of intracellular ROS content labelling with DCFH‐DA and quantitative analysis after 48 h treatment. Scale bars = 250 μm. (C, D) Fe^2+^ content of C2C12 myotubes stained with FerroOrange fluorescent probe up to 48 h of treatment. Scale bars = 250 μm. (E, F) Representative images of SLC7A11, FTL, ACSL4 and GPX4 protein expression and quantification of C2C12 myotubes following treatment up to 48 h. (G, H) QRT‐PCR analysis of changes in MyoG and Myh3 mRNA expression following ferroptosis. (I–K) Representative images of the immunofluorescence (IF) staining of MYHC (green) and nuclei (DAPI, blue), the fusion index, and the diameter of myotubes suggest that ferroptosis inhibits the formation of C2C12 myotubes. Scale bars = 200 μm. (L‐M) Representative images of LCN2 and 24p3R protein expression and quantification of C2C12 myotubes following ferroptosis. (N) QRT‐PCR analysis of LCN2 mRNA expression in C2C12 myotubes following ferroptosis. β‐Actin was used for qRT‐PCR normalisation and GAPDH was used as loading control for the Western blots. ns, not significant, (*p < 0.05, **p < 0.01 and ***p < 0.001) relative to the 0.01% DMSO group, (^#^ *p* < 0.05, ^##^ *p* < 0.01 and ^###^ *p* < 0.001) relative to the era group, as determined by two‐sided Student's t‐test. The data represent the means ± SEMs (*n* = 3 per group).

To precisely understand whether ferroptosis affects myotube formation, C2C12 myotubes were treated with 5 μM erastin either alone or in the presence of 10 μM Fer‐1 after 5 days of differentiation. We measured markers of ferroptosis, as shown by the higher ROS level and Fe^2+^ content in erastin‐treated myotubes than in the 0.01% DMSO group (Figure 3A–D). In addition, the protein expression levels of FTL and ACSL4 were increased under erastin treatment, whereas those of SLC7A11 and GPX4, two essential anti‐lipid peroxidation biomarkers [30] were decreased (Figure 3E,F). These results verified that erastin induced ferroptosis in C2C12 myotubes.

The mRNA expression levels of myogenic marker genes MyoG and myosin heavy chain 3 (Myh3), the terminal differentiation from myoblasts to myotubes, the fusion index [31] and the diameter of myotubes were markedly reduced with erastin, which suggested that C2C12 myotube formation was impaired by erastin‐induced ferroptosis (Figure 3G–K). Notably, LCN2 and its receptor 24p3R expression levels were significantly upregulated in response to erastin, indicating the crucial role of LCN2 in that erastin‐induced ferroptosis inhibits C2C12 myotube formation (Figure 3L–N).

### 3.6 Knockdown of LCN2 Effectively Impairs the Erastin‐Induced Ferroptosis in C2C12 Myoblasts

**FIGURE 4:**
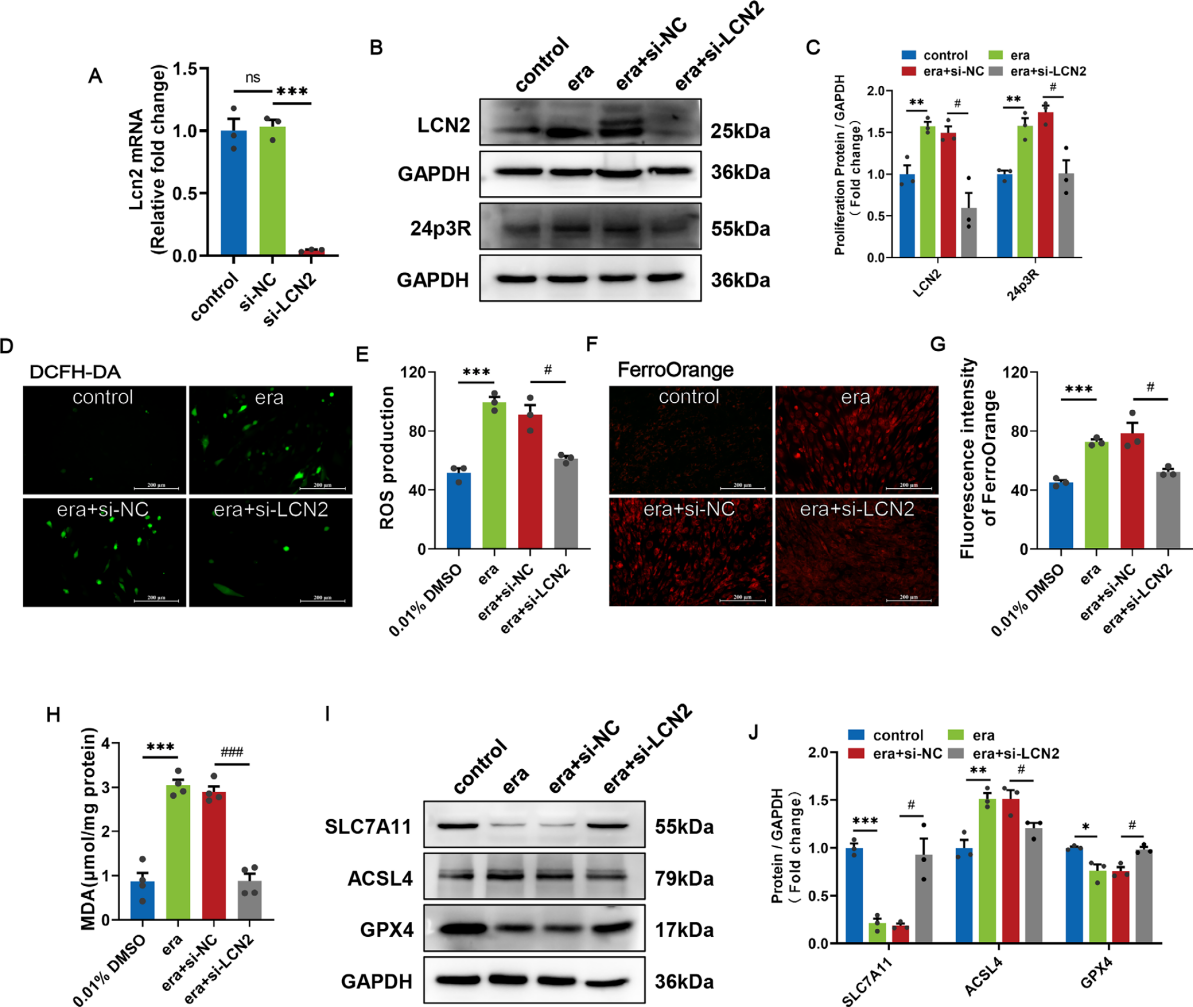
Knockdown of LCN2 effectively impairs the erastin‐induced ferroptosis in C2C12 myoblasts. (A) The knockdown efficiency of specific si‐LCN2 on LCN2 at the mRNA level was detected by qRT‐PCR in C2C12 myoblasts after 48 h. ***p < 0.001 relatives to the si‐NC. (B, C) After transfection with si‐NC or si‐LCN2, C2C12 cells were treated with 5 μM erastin for 48 h. The protein expression levels of LCN2 and 24p3R were verified by western blotting. (D‐E) Representative images of intracellular ROS content labelling with DCFH‐DA and quantitative analysis after transfection of specific si‐RNAs and 48 h treatment with erastin. Scale bars = 200 μm. (F, G) Fe^2+^ content stained with FerroOrange fluorescent probe. Scale bars = 200 μm. (H) Detection of lipid peroxide (MDA) content. (I, J) Representative images of SLC7A11, ACSL4 and GPX4 protein expression and quantification of C2C12 myoblasts after transfection of specific si‐RNAs and 48 h treatment with erastin. β‐Actin was used for qRT‐PCR normalisation and GAPDH was used as loading control for the Western blots. ns, not significant, (*p < 0.05, **p < 0.01 and ***p < 0.001) relative to the 0.01% DMSO, (^#^ *p* < 0.05, ^##^ *p* < 0.01 and ^###^ *p* < 0.001) relative to the era+si‐NC, according to two‐sided Student's t‐test. The data represent the means ± SEMs (*n* = 3 per group).

To investigate the role of LCN2 in the regulation of ferroptosis, we evaluated the effect of LCN2 knockdown on erastin‐stimulated ferroptosis in C2C12 myoblasts. The knockdown efficiency of LCN2 at the mRNA and protein level was examined by qRT‐PCR and Western blots, and the results showed that the knockdown efficiency was good and therefore could be used for subsequent experiments (Figure 4A–C). In addition, si‐LCN2 inhibited the upregulation of 24p3R due to erastin (Figure 4B,C). LCN2 knockdown significantly reduced the levels of ROS, Fe^2+^, and the lipid peroxidation product MDA (Figure 4D–H). In addition, regarding the ferroptosis‐related indicators, the LCN2 knockdown group showed significantly lower levels of ACSL4 protein expression and significantly higher levels of SLC7A11 and GPX4 protein expression compared with the era+si‐NC C2C12 cells group (Figure 4I,J). These results suggest that LCN2 gene deficiency inhibits the occurrence of erastin‐induced ferroptosis in C2C12 myoblasts.

### 3.7 LCN2 Inhibits the Expression of Myogenic Factors by Promoting Ferroptosis

**FIGURE 5:**
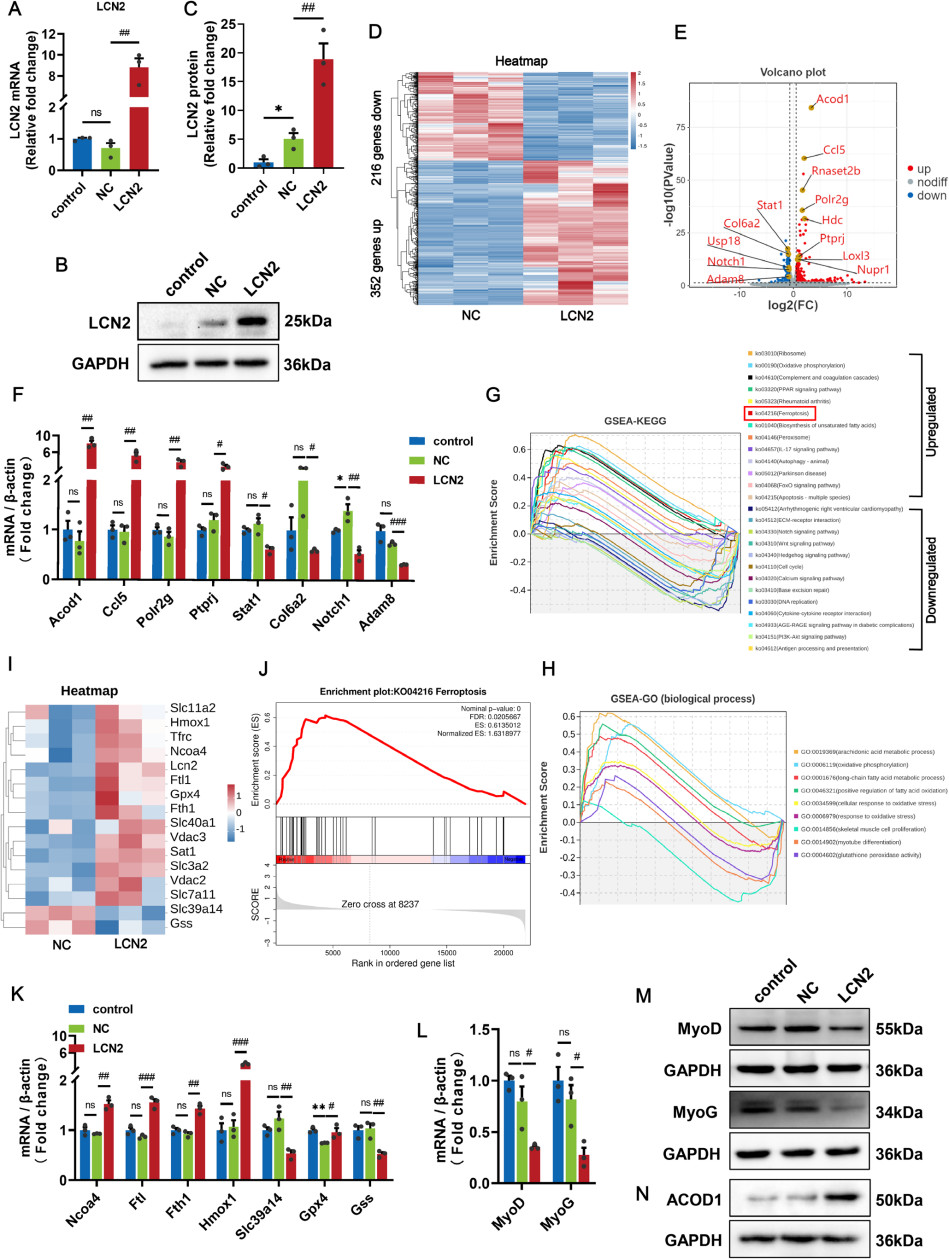
LCN2 Inhibits the expression of myogenic factors by promoting ferroptosis. (A–C) QRT‐PCR and Western blot were used to detect the transfection efficiency of LCN2 at the mRNA and protein levels. (D) RNA sequencing analysis of C2C12 cells overexpressed LCN2. The heatmap represents the result of clustering analysis of DEGs. (E, F) Volcano plot of the DEGs revealed Acod1 was DEG that has the most significant difference, and qRT‐PCR analysis of the partial DEGs mRNA levels was performed. (G) GSEA‐KEGG pathway enrichment analysis of DEGs and ferroptosis signature was enriched after LCN2 overexpression in C2C12 myoblasts. (H) Enriched GSEA‐GO biological processes were identified and listed according to their NES. (I, J) Gene expression heatmap of DEGs related to ferroptosis pathways and the NES showing that ferroptosis was highly upregulated in LCN2 overexpressing myoblasts compared with that in NC myoblasts. (K, L) QRT‐PCR analysis of ferroptosis‐related marker (Ncoa4, Ftl, Fth1, Hmox1, Slc39a14, Gpx4 and Gss) and myogenesis‐related marker (MyoD and MyoG) mRNA levels after LCN2 overexpression in C2C12 myoblasts. (M, N) Western blot was tested for the decline of myogenesis‐related marker (MyoD and MyoG) and the upregulation of ACOD1 protein expression after LCN2 overexpression in C2C12 myoblasts. β‐Actin was used for qRT‐PCR normalisation and GAPDH was used as loading control for the Western blots. ns, not significant, (*p < 0.05 and **p < 0.01) relative to the control, (^#^ *p* < 0.05, ^##^ *p* < 0.01 and ^###^ *p* < 0.001) relative to the NC, according to two‐sided Student's t‐test. The data represent the means ± SEMs (*n* = 3 per group).

To systematically investigate whether LCN2 influences the regeneration of skeletal muscle by mediating ferroptosis and its specific mechanism, mouse pcDNA3.1 or pcDNA3.1 + ‐LCN2 vectors were transfected into C2C12 myoblast cells. After 48 h, we extracted RNA and then conducted RNA sequencing and transcriptomic analysis. We examined LCN2 overexpression efficiency via qRT‐PCR and Western blot assays, which showed a good overexpression (Figure 5A–C). The RNA sequencing results showed that LCN2 increased the expression of 352 genes and decreased that of 216 genes in C2C12 cells (Figure 5D; Table S3). Importantly, the gene that was significantly upregulated in DEGs and showed the most significant difference was Acod1. Among the other DEGs, the RNA sequencing results also showed that LCN2 upregulated the genes encoding Ccl5, Rnaste2b, Polr2g, Hdc, Ptprj, Loxl3 and Nupr1 and repressed the genes encoding STAT1, Col6a2, Usp18, Notch1 and Adam8 (Figure 5E), and these data were also verified by qRT‐PCR (Figure 5F). GSEA via the KEGG database showed DEGs were enriched in pathways such as ‘oxidative phosphorylation’, ‘FoxO signalling pathway’ and ‘ferroptosis’ (Figure 5G). GO biological process terms were enriched in dysregulation of redox equilibrium and metabolic homeostasis of skeletal muscle cells, which were represented by upregulated genes about oxidative phosphorylation, response to oxidative stress, arachidonic acid and long‐chain fatty acid metabolic processes, while the downregulated genes were involved in glutathione peroxidase activity, skeletal muscle cell proliferation and myotube differentiation (Figure 5H). Considering that arachidonic acid is one of the main substrates of ferroptosis‐related lipid peroxidation [32, 33], this work focused specifically on ferroptosis‐related genes. Thus, we verified ferroptosis‐related genes in RNA‐seq data by qRT‐PCR analysis, and the results showed that LCN2 increased the mRNA expression of haeme oxygenase 1 (Hmox1), Ncoa4, Ftl, ferritin heavy chain (Fth1) and Gpx4, decreased the expression of solute carrier family 39 member 14 (Slc39a14), and glutathione synthetase (Gss). The normalised enrichment score (NES) was 1.6 > 0, implying upregulation of the ferroptosis signalling pathway (Figure 5I–K). Further, our tests revealed the decrease of myogenesis‐related indicators MyoD and MyoG mRNA and protein levels, suggesting that LCN2 inhibited the expression of myogenic factors by activating the ferroptosis signature (Figure 5L,M and S3A). The upregulation of ACOD1 protein level was verified as it is localised in the mitochondria, which play an important role in ferroptosis (Figure 5N and S3B) [34]. Collectively, these findings imply that LCN2 inhibits skeletal muscle cell growth by promoting ferroptosis, which may be related to the upregulation of ACOD1 by LCN2.

### 3.8 ACOD1 Acts as an Effector of LCN2 in Promoting Mitochondria‐Associated Ferroptosis

**FIGURE 6:**
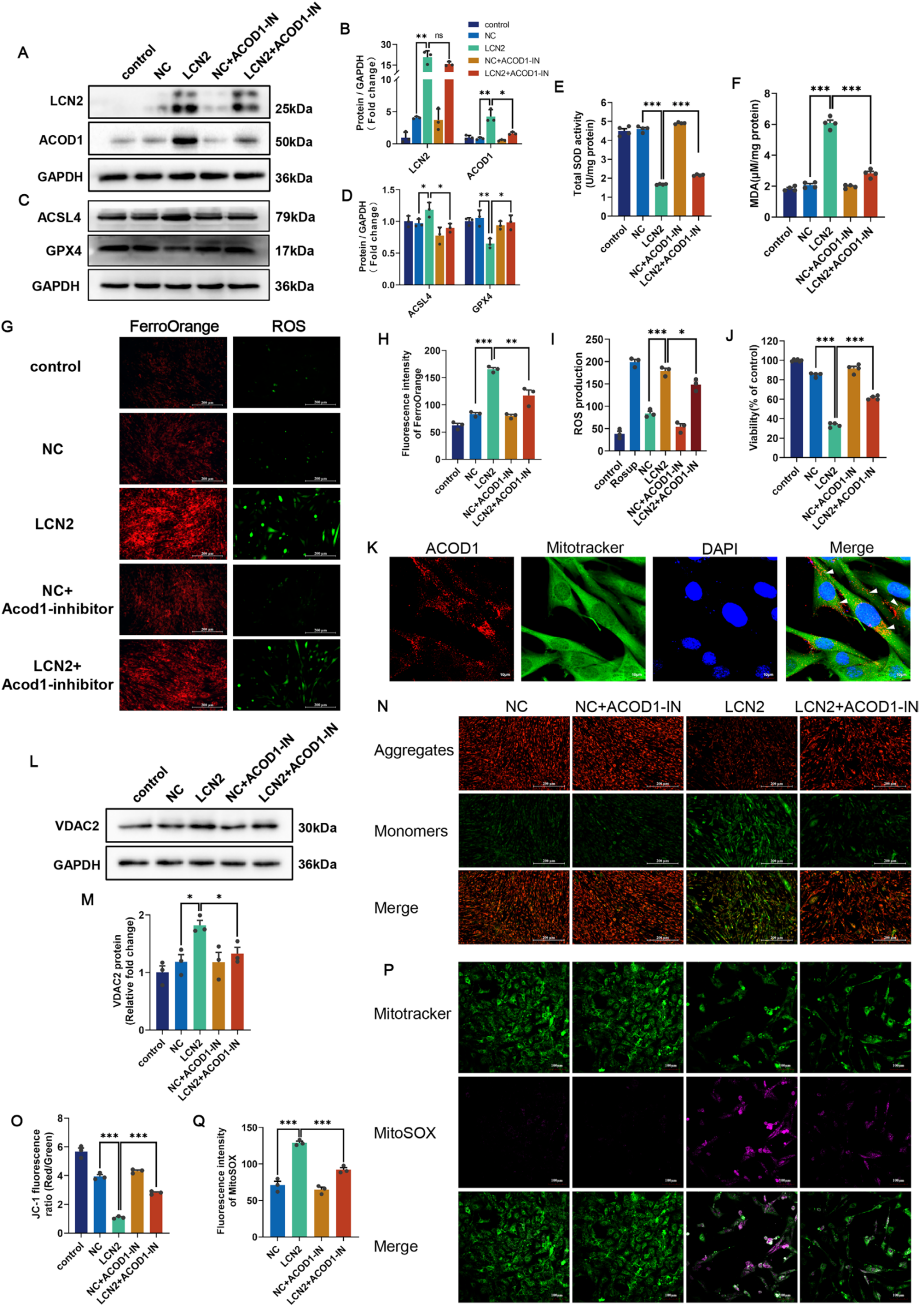
ACOD1 acts as an effector of LCN2 in promoting mitochondria‐associated ferroptosis. (A, B) Western blots were used to verify the protein expression level of LCN2 and ACOD1 after overexpression of LCN2 with or without 20 μM ACOD1‐IN after 48 h of treatment. (C, D) Representative images of ACSL4 and GPX4 protein expression and analysis of C2C12 myoblasts after 48 h of treatment. (E, F) Activity of SOD and lipid peroxide (MDA) content were detected. (G–I) Representative images show the amount of Fe^2+^ and intracellular ROS content in the indicated cells and their quantitative analysis. Scale bars = 200 μm. (J) Cell viability of C2C12 myoblasts after overexpression of LCN2 either alone or in the presence of 20 μM ACOD1‐IN after 48 h of treatment. (K) Subcellular localisation of IRG1 was analysed by ICF in C2C12 cells after stained with 150 nM mitotracker. Scale bars = 10 μm. (L, M) Representative Western blot image of VDAC2 and its quantification. (N‐O) JC‐1 dyeing was used to evaluate the effect of LCN2 stimulation on MMP, and this effect was largely reversed by ACOD1‐IN. Scale bars = 200 μm. (P‐Q) Representative images and quantitative analysis of mitoROS stained with MitoSOX to examine the degree of lipid peroxidation driven by mitochondria. Scale bars = 100 μm. GAPDH was used as a loading control for the Western blots. ns, not significant, *p < 0.05, **p < 0.01 and ***p < 0.001, two‐sided Student's t‐test. The data represent the means ± SEMs (*n* = 3 per group).

To further corroborate the relationship between the LCN2‐ACOD1 pathway and ferroptosis, 20 μM ACOD1‐IN was added to C2C12 myoblasts after overexpressing LCN2. LCN2 protein expression remained elevated even in ACOD1‐IN treated myoblasts, and the increase in ACOD1 protein expression was reduced by the ACOD1‐IN (Figure 6A,B). Overexpression of LCN2 indeed aggravated ferroptosis, as evidenced by the increased protein expression level of ACSL4 and the decreased protein expression level of GPX4 (Figure 6C,D). Additionally, LCN2 downregulated the activity of SOD (Figure 6E), increased MDA content (Figure 6F), Fe^2+^(Figure 6G,H) and intracellular ROS (Figure 6G–I), all of which were reversed by the ACOD1‐IN. Furthermore, ACOD1‐IN alleviated the inhibitory effect of LCN2 on cell viability (Figure 6J). Taken together, these results suggest that LCN2 exacerbates ferroptosis by upregulating ACOD1, thereby attenuating the myogenic potential of myoblasts.

It has been reported that ACOD1 is localised in mitochondria and this has also been confirmed in our research (Figure 6K). Mitochondria are also the main source of ROS to drive ferroptosis [35]. Ferroptosis triggered by LCN2‐ACOD1 signalling is hypothesised to be associated with mitochondrial dysfunction. The protein levels of voltage‐dependent anion channel 2 (VDAC2), a key component involved in mitochondrial material transport [36], were significantly increased after the overexpression of LCN2 (Figure 6L,M). This effect was largely reversed by ACOD1‐IN, which means ACOD1 provokes dysregulation of the metabolism of mitochondrial substances as well as a disturbance of the redox balance. Next, we determined the effects of LCN2‐stimulated C2C12 myoblasts on MMP and superoxide production via JC‐1 and MitoSOX, respectively (Figure 6N–Q). It was found that the reduction in the ratio of JC‐1 aggregates/monomers was significantly attenuated, indicating a reduction in MMP in response to LCN2‐ACOD1 signalling. Similarly, the accumulation of lipid peroxidation products in mitochondria was lower with the addition of ACOD1‐IN compared to the overexpression of LCN2. These data suggest that ACOD1 acts as an effector of LCN2 to induce mitochondrial dysfunction‐mediated ferroptosis in C2C12 myoblasts.

**FIGURE 7:**
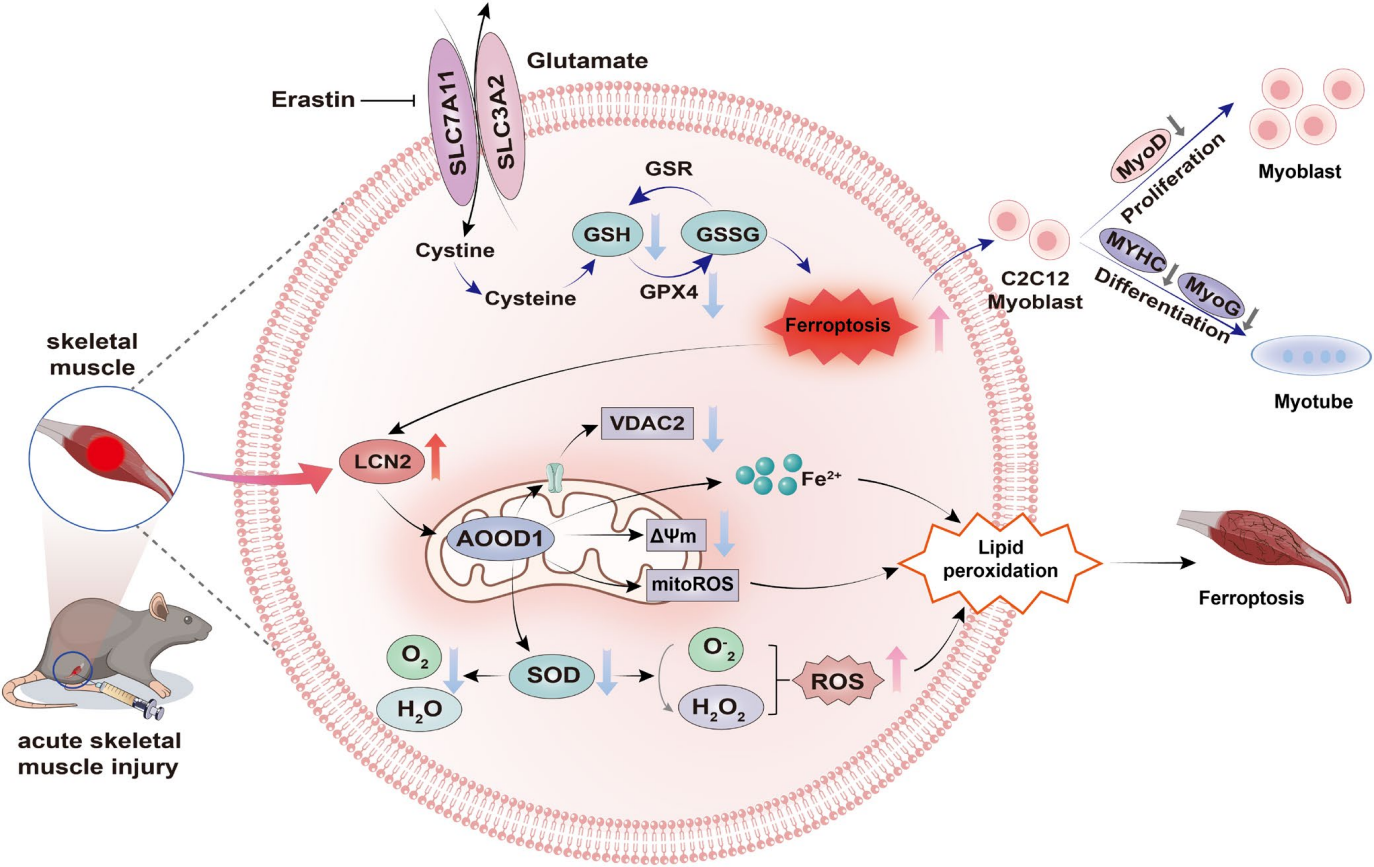
ACOD1 acts as an effector of LCN2 in promoting mitochondria‐associated ferroptosis. Hypothetical diagram of how LCN2 affects post‐injury regeneration of skeletal muscle by mediating ferroptosis. When the secreted protein LCN2 is abundantly expressed in skeletal muscle cells, the expression of the mitochondrial protein ACOD1 is significantly upregulated, which triggers mitochondrial dysfunction‐associated ferroptosis, and subsequently adversely affects skeletal muscle regeneration.

In summary, these data strongly support that LCN2 promotes mitochondrial dysfunction‐associated ferroptosis through the upregulation of ACOD1. Inhibition of ACOD1 alleviated LCN2‐induced ferroptosis and improved the regeneration process of skeletal muscle cells (Figure 7).

## 4 Discussion

Programmed cell death, such as apoptosis, autophagy and necrosis, is caused by external factors in response to skeletal muscle damage. These processes coexist in injured/regenerating muscles, including those of patients with neuromuscular diseases, such as inflammatory myopathies, dystrophies, metabolic and mitochondrial myopathies, and drug‐induced myopathies [3]. Along with these findings, a unique form of programmed cell death, ferroptosis, is gradually being revealed relative to the processes of damage repair and regeneration in skeletal muscle. Deficiency of Tfr1 in satellite cells (SCs) from the aged skeletal muscle of rodents and human sarcopenia disrupts skeletal muscle regeneration through the activation of ferroptosis, a process accompanied by a functional switch of TFR1‐SLC39a14‐iron axis [9]. The downregulation of cystathionine gamma‐lyase/hydrogen sulphide (CSE/H2S) signalling would contributes to ferroptotic cell death and enhances global protein acetylation in mouse skeletal muscles under aging or injury conditions [37]. The expression of ferroptosis‐related markers ACSL4 and Hmox‐1 was increased in the CTX‐induced muscle regeneration model, and subsequently, the expression of muscle regeneration‐related genes MyoD and MyoG was inhibited by ferroptosis inhibitors deferoxamine (DFO) and UAMC‐3203, indicating that ferroptosis may mediate muscle regeneration [8]. Consistent with the above results, our study revealed ferroptosis characteristics, such as morphological changes in mitochondria, increases in the contents of serum Fe^2+^ and MDA in GAS, and increases of NCOA4, Ftl and ACSL4 expression on 1 D and 3 D during the injury stage of skeletal muscle, whereas GPX4 expression was decreased. One additional point to be noted is that studies have examined and found that compared to other commonly used internal control genes such as β‐actin and GAPDH, N‐acylneuraminate cytidylyltransferase (CMAS) shows relatively stable expression during muscle regeneration. Therefore, we adopted the CMAS recommended as the internal control gene for gene expression analysis during muscle regeneration [38].

Ferroptosis is a regulated form of cell death that can be regulated, for example, by inhibiting system XC^−^. Erastin, a system XC^−^ inhibitor and ferroptosis activator, is metabolically stable and suitable for use both in vivo and in vitro [39]. The C2C12, U57810 and U23674 mouse lines are sensitive to erastin, which dose‐dependently reduces the viability of the cells mentioned above [40]. This is coherent with our CCK‐8 results after different concentrations of erastin were applied to C2C12 myoblasts. As a ferroptosis activator, system XC^−^ activity is inhibited by binding to SLC7A11 [41], cystine transport is impaired to reduce GPX4 expression and GSH synthesis, and ultimately, cells cannot scavenge lipid peroxides timely, leading to the cell membrane damage [42]. Thus, we detected the upregulation of Fe^2+^, MDA and ROS contents, in addition to the downregulation of SLC7A11 and GPX4 and upregulation of FTL and ASCL4 expression in C2C12 myoblasts and myotubes treated with erastin. In addition, ferroptosis triggered by erastin inhibited C2C12 myoblast proliferation and myotube formation, which is consistent with previous findings [43].

In this study, LCN2 expression was significantly upregulated during the occurrence of erastin‐induced ferroptosis of C2C12 myoblasts and myotubes, and LCN2 expression was sharply elevated during the early injury, suggesting that LCN2 was involved in regulating skeletal muscle regeneration by mediating ferroptosis. Under physiological and inflammatory conditions, LCN2 has been identified as an iron‐regulatory protein. In prokaryotes, LCN2 restrains bacterial siderophores from acquiring iron, thus inhibiting bacterial growth [44]. In mammals, a study using a mouse model of leptomeningeal metastasis showed that cancer cells use LCN2 to collect iron [45]. In addition, several studies have proved that LCN2 may represent a valuable target for the treatment of a variety of diseases by mediating ferroptosis. LCN2 promotes glutamate‐induced mitochondria‐mediated ferroptosis through activation of the NF‐κB/STAT3 axis, thus confirming that LCN2, a key gene for ferroptosis, plays an important role in the development of hypoxic–ischemic brain damage (HIBD) [46]. In experimental models of lung cancer cachexia, a significant increase in the number of tissue‐infiltrating neutrophils was detected, and these cells secreted high levels of LCN2 to promote ferroptosis and tissue wasting [13]. Evidence from different studies is consistent, including our findings that enhanced expression levels of LCN2 were detected in erastin‐induced ferroptosis models of hepatocellular carcinoma and pancreatic ductal adenocarcinoma cells [47, 48]. In recent years, the role of LCN2, an acute‐phase protein in skeletal muscle metabolism has been revealed. Compared with the wild‐type mice, satellite cell activation and skeletal muscle regeneration are significantly impaired in LCN2^−/−^ mice [16]. However, it is not clear whether LCN2 regulates skeletal muscle myogenesis by mediating ferroptosis and its specific mechanism. Here, RNA sequencing was performed after LCN2 was overexpressed in C2C12 myoblasts. Consistent with the results after GAS injury, sequencing revealed that LCN2 could promote ferroptosis by upregulating NCOA4 to promote an increase of free iron and decrease the level of Gss, which catalyses glutathione synthesis, and that the upregulation of Gpx4 may be a resistance response to negative signalling.

Our work reveals that ACOD1 is an effector of LCN2 that promotes ferroptosis and thus affects the regeneration of skeletal muscle. ACOD1 is thought to be an enzyme that is active in itaconate metabolism and is found in mitochondria mainly [18, 49]. Abnormal ACOD1 expression is associated with inflammatory responses, antibacterial processes, tumourigenesis, neurodegenerative changes and embryo implantation [18, 50, 51, 52]. ACOD1 mediates oxidative stress by promoting intracellular ROS and mitoROS production. ROS are catalysed by excess Fe^2+^ to oxidise polyunsaturated fatty acids (PUFAs) on lipid membranes to produce lipid peroxides, which in turn trigger ferroptosis [53]. It has been reported that ACOD1 regulates the ferroptosis to affect the breast cancer metastasis [54], which is consistent with our finding that ferroptosis is downregulated in C2C12 myoblasts treated with ACOD1‐IN after LCN2 overexpression. Under stress conditions, especially inflammatory stimulation, ACOD1 expression is upregulated by macrophages, monocytes and dendritic cells in the innate immune system. Moreover, cytokines (e.g., interferon beta 1 [IFNB1], interferon‐gamma [IFNG]) and small molecule drugs (e.g., chemical inducer of HMOX1) can stimulate the expression of ACOD1 [55]. Coincidentally, our transcriptome sequencing results revealed that HMOX1 was significantly upregulated after LCN2 overexpression, but the specific relationships among LCN2, HMOX1 and ACOD1 in regulating skeletal muscle regeneration after injury need to be explored further.

Ferroptosis and mitochondrial dysfunction are inextricably linked. Skeletal muscle is rich in mitochondria, which actively participate in different types of regulated cell death mechanisms, including apoptosis, pyroptosis, necroptosis, ferroptosis and autophagy [56]. The main mitochondrial pathways engaged in ferroptosis are multiple; for example, that mitochondria participate in mitoROS production, iron accumulation, lipid and amino acid metabolism, glutaminolysis, redox status regulation and cell antioxidant capacity [34]. A recent study proposed that mitochondria are indispensable for erastin‐ or cystine starvation‐induced ferroptosis [57]. These findings confirmed that LCN2 is involved in the inhibition of myogenesis due to erastin‐induced ferroptosis, and we found that MMP was reduced and that mitoROS accumulated when LCN2‐ACOD1 signalling was upregulated. In addition, VDAC2 is involved in redox regulation [58] in addition to transporting metabolites [36], thus, the upregulation of VDAC2 implies that LCN2‐ACOD1 triggers an adaptive response of mitochondria to oxidative stress.

In conclusion, our results confirm that the expression level of LCN2 is significantly correlated with skeletal muscle injury, and more importantly, we found for the first time that LCN2‐ACOD1 signalling affects skeletal muscle regeneration by mediating mitochondria‐associated ferroptosis. Taken together, the present study elucidates a new mechanism of skeletal muscle regeneration post injury, which sheds light on a new target for therapeutic strategies against muscle‐related diseases.

## Author Contributions

Haidong Wang and Juan Wang conceived the research concept and design; Xiaojing Hao, Hongwei Shi, Di Wu, Rui Liang, Tong Zhao, Wen Sun and Yue Wang performed the experiments; Xiaojing Hao and Hongwei Shi collected and conducted statistical analysis of the data; Jiayin Lu and Haidong Wang supervised the study; Xiuju Yu, Xiaomao Luo, Yi Yan and Juan Wang provided technical assistance; Xiaojing Hao, Jiayin Lu and Haidong Wang wrote and revised the manuscript; Haidong Wang provided funds. All authors read and approved the final manuscript.

## Ethics Statement

The animal work was approved by the Institutional Animal Care and Use Committee of Shanxi Agricultural University [SXAU‐EAW‐2023 M.ZZ.004009196].

## Conflicts of Interest

The authors declare no conflicts of interest.

## Supporting information


**Figure S1.** Effects of BaCl_2_ on skeletal muscle injury, repair and regeneration in mice. (A) Representative images of H&E staining of GAS collected immediately before and at 1, 3, 5, 7 and 9 days after BaCl_2_ injection in mice (yellow arrows mark damaged myocytes, red arrows mark inflammatory cells and black arrows mark regenerating myofibers). Scale bars = 200 μm. (B, D) The distribution of cross‐sectional area (CSA) of myofiber in GAS from mice before or after BaCl_2_ induced injury, the mean area of muscle fibres and the percentage of myofibers containing two or more centrally located nuclei. (E, F) Representative images of Masson staining (green arrows indicate collagen fibres) and analysis of collagen volume fraction (CVF). Scale bars = 200 μm. (G) QRT‐PCR analysis of Pax7, MyoD and MyoG mRNA expression in GAS from mice before or after BaCl_2_ induced injury. (H, I) Representative western blots images of Pax7, MyoD and MyoG expression and quantification of GAS from mice before or after BaCl_2_ induced injury. Cmas was used for qRT‐PCR normalisation and CMAS was used as loading control for the Western blots. ns, not significant, (*p < 0.05, **p < 0.01 and ***p < 0.001) relative to the control, according to two‐sided Student's t‐test. The data represent the means ±SEMs (*n* = 3–4 per group).

**Figure S2.** LCN2 is a crucial factor in attenuating myogenic differentiation of C2C12 myoblasts through ferroptosis. Differentiating C2C12 myoblasts were treating with 5 μM erastin with or without inhibitors Fer‐1 for 5 days. (A–C) Representative images of intracellular ROS content labelling with DCFH‐DA and quantitative analysis after 5 days treatment. Scale bars = 200 μm. (B, D) Fe^2+^ content of C2C12 myotubes stained with FerroOrange fluorescent probe. Scale bars = 200 μm. (E) Detection of lipid peroxide (MDA) content. (F, G) Representative images of SLC7A11, ACSL4 and GPX4 protein expression and quantification of differentiating C2C12 myoblasts after 5 days treatment. (H, I) Representative IF staining images of MYHC (red) and nuclei (DAPI, blue) and the statistics of total cell nuclei in differentiating C2C12 myoblasts on 1 day, 3 days and 5 days differentiation. Scale bars = 200 μm. (J) Quantification of the fusion index, defined as the percentage of nuclei within MYHC‐positive multinucleated myotubes. (K) The diameter of myotubes after 5 days treatment. (L, M) The protein expression level of LCN2 was detected by Western blots after 5 days differentiation. GAPDH was used as loading control for the Western blots. ns, not significant (*p < 0.05, **p < 0.01 and ***p < 0.001) relative to the 0.01% DMSO group (#*p* < 0.05, ##*p* < 0.01 and ###*p* < 0.001) relative to the era group, as determined by two‐sided Student's t‐test. The data represent the means ± SEMs (*n* = 3 per group).

**Figure S3.** LCN2 inhibits the expression of myogenic factors by promoting ferroptosis. (A) Western blot analysis of myogenesis‐related marker (MyoD and MyoG) after LCN2 overexpression in C2C12 myoblasts. (B) Western blot analysis was verified the upregulation of ACOD1 protein expression after LCN2 overexpression in C2C12 myoblasts. GAPDH was used as loading control for the Western blots. ns, not significant (#*p* < 0.05 and ##*p* < 0.01) relative to the NC group, according to two‐sided Student's t‐test. The data represent the means ± SEMs (*n* = 3 per group).

**Table S1.** Antibodies used for Western blot analysis.
**Table S2.** Sequence of specific primers for target genes.
**Table S3.** Significantly differentially expressed gene sets identified after in vitro LCN2 overexpression.

## Data Availability

The data that support the findings of this study are available from the corresponding author upon reasonable request.
