# Supplementary figures and images for: LCN2‐ACOD1 Signalling Affects the Post‐Injury Regeneration of Skeletal Muscle Through Mediating Ferroptosis
Source: Cell Prolif. 2025 Sep 22;59(4):e70130. doi: 10.1111/cpr.70130 (PMC13051941; doi:10.1111/cpr.70130)

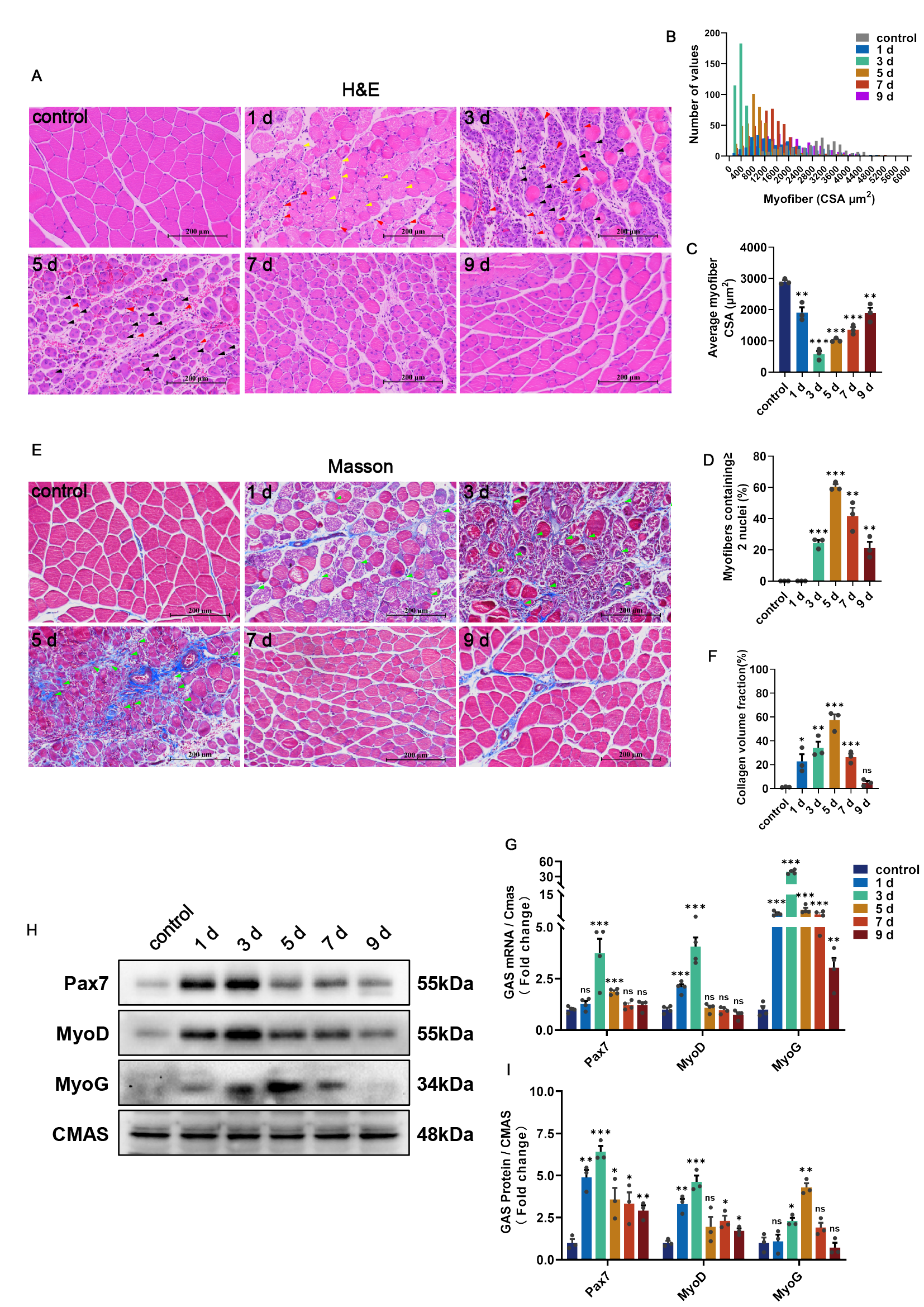

Supplement: Supplementary file 1 — Figure S1. Effects of BaCl2 on skeletal muscle injury, repair and regeneration in mice. (A) Representative images of H&E staining of GAS collected immediately before and at 1, 3, 5, 7 and 9 days after BaCl2 injection in mice (yellow arrows mark damaged myocytes, red arrows mark inflammatory cells and black arrows mark regenerating myofibers). Scale bars = 200 μm. (B, D) The distribution of cross‐sectional area (CSA) of myofiber in GAS from mice before or after BaCl2 induced injury, the mean area of muscle fibres and the percentage of myofibers containing two or more centrally located nuclei. (E, F) Representative images of Masson staining (green arrows indicate collagen fibres) and analysis of collagen volume fraction (CVF). Scale bars = 200 μm. (G) QRT‐PCR analysis of Pax7, MyoD and MyoG mRNA expression in GAS from mice before or after BaCl2 induced injury. (H, I) Representative western blots images of Pax7, MyoD and MyoG expression and quantification of GAS from mice before or after BaCl2 induced injury. Cmas was used for qRT‐PCR normalisation and CMAS was used as loading control for the Western blots. ns, not significant, (*p < 0.05, **p < 0.01 and ***p < 0.001) relative to the control, according to two‐sided Student's t‐test. The data represent the means ±SEMs (n = 3–4 per group). [file CPR-59-e70130-s001.tif]

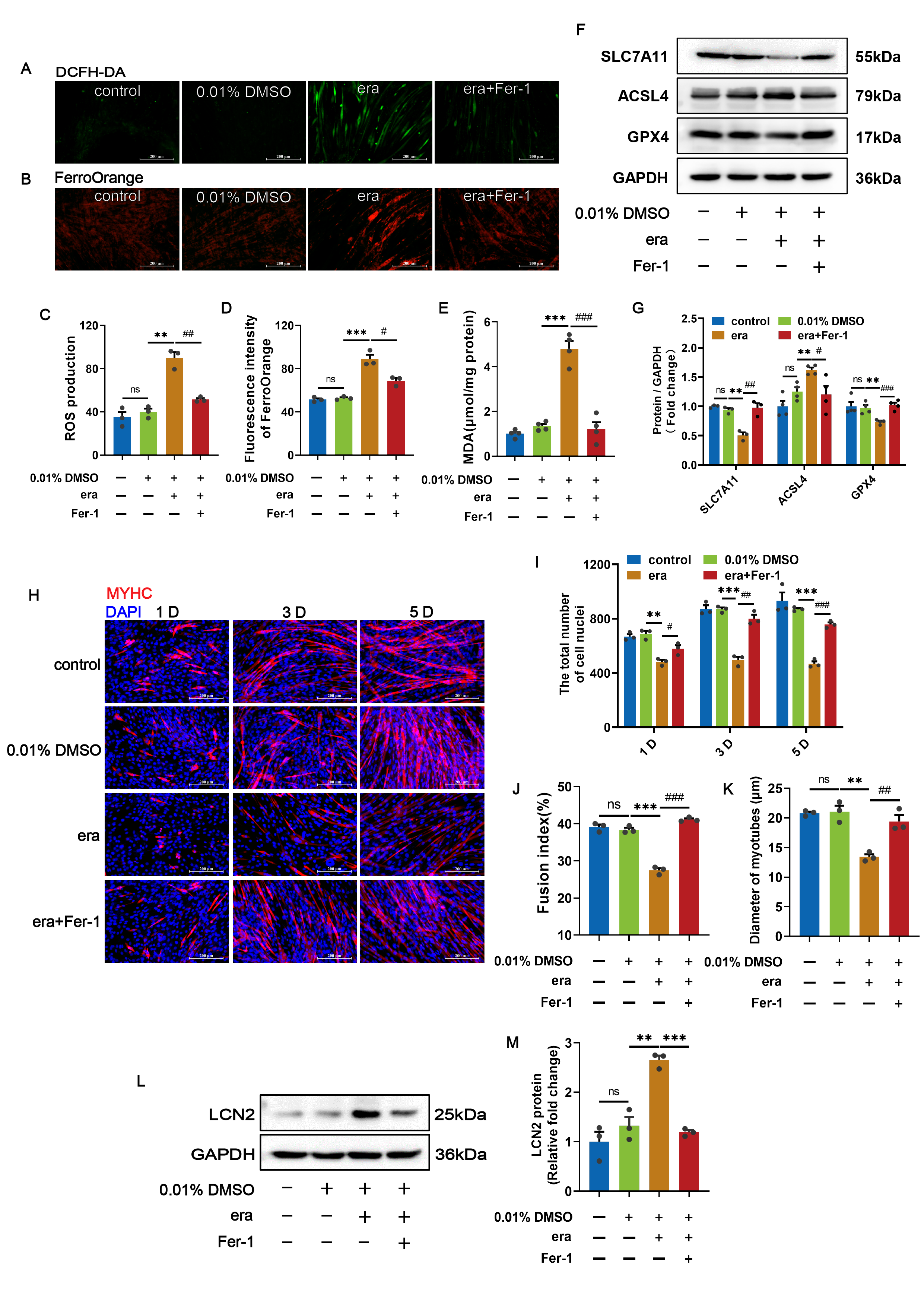

Supplement: Supplementary file 2 — Figure S2. LCN2 is a crucial factor in attenuating myogenic differentiation of C2C12 myoblasts through ferroptosis. Differentiating C2C12 myoblasts were treating with 5 μM erastin with or without inhibitors Fer‐1 for 5 days. (A–C) Representative images of intracellular ROS content labelling with DCFH‐DA and quantitative analysis after 5 days treatment. Scale bars = 200 μm. (B, D) Fe2+ content of C2C12 myotubes stained with FerroOrange fluorescent probe. Scale bars = 200 μm. (E) Detection of lipid peroxide (MDA) content. (F, G) Representative images of SLC7A11, ACSL4 and GPX4 protein expression and quantification of differentiating C2C12 myoblasts after 5 days treatment. (H, I) Representative IF staining images of MYHC (red) and nuclei (DAPI, blue) and the statistics of total cell nuclei in differentiating C2C12 myoblasts on 1 day, 3 days and 5 days differentiation. Scale bars = 200 μm. (J) Quantification of the fusion index, defined as the percentage of nuclei within MYHC‐positive multinucleated myotubes. (K) The diameter of myotubes after 5 days treatment. (L, M) The protein expression level of LCN2 was detected by Western blots after 5 days differentiation. GAPDH was used as loading control for the Western blots. ns, not significant (*p < 0.05, **p < 0.01 and ***p < 0.001) relative to the 0.01% DMSO group (#p < 0.05, ##p < 0.01 and ###p < 0.001) relative to the era group, as determined by two‐sided Student's t‐test. The data represent the means ± SEMs (n = 3 per group). [file CPR-59-e70130-s004.tif]

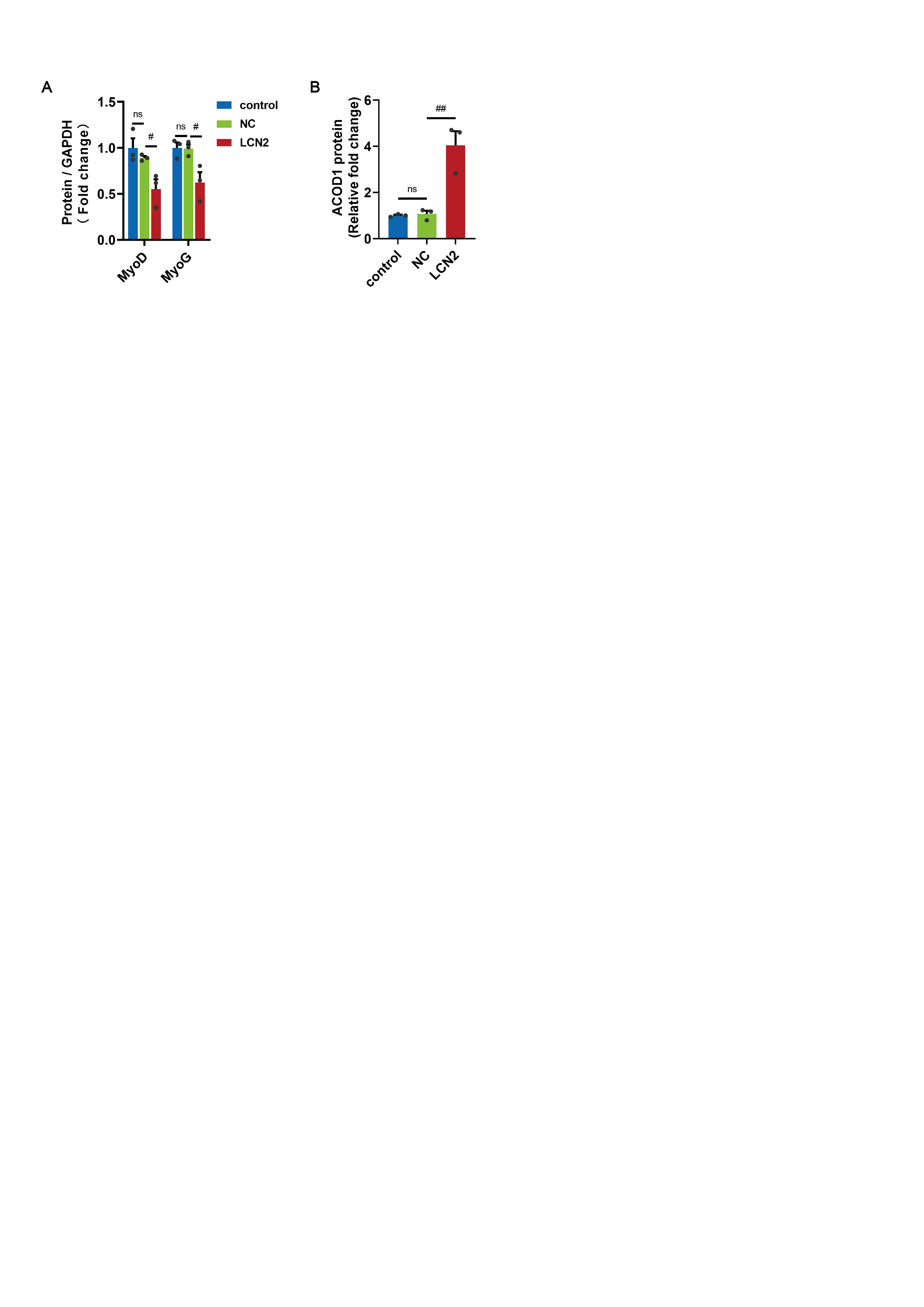

Supplement: Supplementary file 3 — Figure S3. LCN2 inhibits the expression of myogenic factors by promoting ferroptosis. (A) Western blot analysis of myogenesis‐related marker (MyoD and MyoG) after LCN2 overexpression in C2C12 myoblasts. (B) Western blot analysis was verified the upregulation of ACOD1 protein expression after LCN2 overexpression in C2C12 myoblasts. GAPDH was used as loading control for the Western blots. ns, not significant (#p < 0.05 and ##p < 0.01) relative to the NC group, according to two‐sided Student's t‐test. The data represent the means ± SEMs (n = 3 per group). [file CPR-59-e70130-s003.tif]
